# Novel Surrogates for Membrane Fouling and the Application of Support Vector Machine in Analyzing Fouling Mechanism

**DOI:** 10.3390/membranes11120990

**Published:** 2021-12-18

**Authors:** Xianghao Meng, Fukuan Wang, Shujuan Meng, Rui Wang, Zhongyuan Mao, Yue Li, Meifeng Yu, Xuye Wang, Qian Zhao, Linyan Yang

**Affiliations:** 1 School of Space and Environment, Beihang University, Beijing 100191, China; sy1930212@buaa.edu.cn (X.M.); rui_wang@buaa.edu.cn (R.W.); maozhongyuan@buaa.com (Z.M.); 18377207@buaa.edu.cn (Y.L.); 18377318@buaa.edu.cn (M.Y.); wang_xy@buaa.edu.cn (X.W.); 2 College of Mechanical Engineering, Guangxi University, Nanning 530004, China; wfukuan@163.com; 3 School of Municipal and Environmental Engineering, Shandong Jianzhu University, Jinan 250101, China; zhaoqian@sdjzu.edu.cn; 4 School of Resources and Environmental Engineering, East China University of Science and Technology, Shanghai 200237, China; lyyang@ecust.edu.cn

**Keywords:** membrane fouling, extracellular polymeric substances, surrogates of foulants, support vector machine

## Abstract

It is difficult to recognize specific fouling mechanisms due to the complexity of practical feed water, thus the current studies usually employ foulant surrogates to carry out research, such as alginate and xanthan gum. However, the representativeness of these surrogates is questionable. In this work, the classical surrogates (i.e., alginate and xanthan gum) were systematically studied, and results showed that they behaved differently during filtration. For the mixture of alginate and xanthan gum, both filtration behaviors and adsorption tests performed by quartz-crystal microbalance with dissipation monitoring (QCM-D) indicated that alginate plays a leading role in fouling development. Furthermore, by examining the filtration behaviors of extracellular polymeric substances (EPS) extracted from practical source water, it turns out that the gel layer formation is responsible for EPS fouling, and the properties of gel layer formed by EPS share more similarities with that formed from pectin instead of alginate. In addition, with the use of experimental data sets extracted from this study and our previous studies, a modeling method was established and tested by the support vector machine (SVM) to predict complex filtration behaviors. Results showed that the small differences of fouling mechanisms lying between alginate and pectin cannot be recognized by Hermia’s models, and SVM can show a discrimination as high as 76.92%. As such, SVM may be a powerful tool to predict complex filtration behaviors.

## 1. Introduction

As an advanced water treatment technology, membrane separation can be performed separately or in combination with other processes to offer high-quality effluent [1,2]. However, membrane fouling, which is an inevitable result of separating contaminants from water, has, to a considerable extent, limited the application of membrane technology in a wider scope [3]. According to the composition of the dominant foulants of membrane, organic matter with high viscosity and high C/N ratio plays a significant role in fouling development [4,5]. Recently, polysaccharide has become regarded as one of the main causes of membrane fouling because it is much larger than humic acids and protein, and, more importantly, it possesses gelling properties which enables it to work as the skeleton of fouling layer [6,7]. Recently, the transparent exopolymer particles (TEP) forming from acidic polysaccharides, have shown their effect on membrane fouling [8]. TEP has been found in almost all feed water to membrane systems, and the growing evidence has shown its essential impact on membrane fouling [9,10], which, in turn, emphasizes the significance of polysaccharide substance in membrane fouling. However, there is still a knowledge gap between the fouling propensities of polysaccharides and extracellular polymeric substances (EPS). As such, the divergence of polysaccharide fouling and EPS fouling should be explored, and the representativeness of polysaccharide fouling in the interpretation of practical fouling problems should be addressed.

Polysaccharide fouling has been widely studied, and, in these studies, surrogate foulants are commonly employed. Alginate and xanthan gum are the most popular surrogates in fouling studies. However, due to the divergence observed in between the abundant organic foulants and surrogates, neither alginate nor xanthan gum can be viewed as perfect examples to represent all polysaccharides in fouling studies. Recently, there are some reports showing that the representativeness of these surrogates in fouling properties is questionable when considering real foulants in practical feed water [11,12]. In order to solve this problem, our previous studies contained various polysaccharide substances involved in fouling analysis, in an effort to establish a basic database reflecting the fouling properties of complicated foulants in feed water [13]. It has been shown that the fouling mechanisms of diverse polysaccharides can be divided into four categories: standard blocking, complete blocking, intermediate blocking, and gel layer [8,13,14]. However, the dominant fouling type should be further identified. Furthermore, EPS extracted from practical feed water can be employed to investigate the fouling problems.

Fouling modeling is an effective tool in analyzing filtration data and revealing fouling mechanisms. There are two types of fouling models, and these include the Hermia mathematical models, and the empirical ANN model [15]. Usually, the traditional mathematical models are established based on many assumptions, which weakens their analytical precision. In the empirical model, ANN is popular, but it has some problems (i.e., does not provide information about the relative importance of the various parameters, and it needs a large database). Support vector machine (SVM) is a novel type of empirical model, which has shown excellent performance in limited samples and non-linear function. Therefore, SVM could probably provide a good analysis of the non-linear relationship between the operation parameters (foulant types, pH, concentration, etc.) and the output product of the fouling mechanism [16]. Based on this, membrane fouling mechanism may be identified by the collection of sample data and the screening of characteristic parameters. As such, in this study, a novel fouling model based on SVM analysis was proposed to identify the fouling mechanisms.

## 2. Materials and Methods

### 2.1. Experiment Materials and Preparation of Solution

Commercial polyethersulfone ultrafiltration membrane (20 kDa, AMFOR INC, Beijing, China) was used throughout the experiment; its effective surface area was controlled at 42 cm^2^. Alginate (Tianjin Jingke Fine Chemical Research Institute, Tianjin, China), pectin (citrus peels, Macklin, Shanghai, China), and xanthan gum (Solarbio), were prepared with the concentrations found in Table 1. Calcium chloride (CaCl_2_), magnesium chloride (MgCl_2_), and sodium-dodecyl-sulfate (SDS) were obtained from Macklin (Shanghai, China). The 1 mol·L^−1^ NaOH (AR) and formaldehyde (AR) were obtained from Beijing Chemical Works (Beijing, China). Ultrapure water was obtained from a Milli-Q ultrapure water purification system (Millipore Simplicity, Molsheim, France).

### 2.2. Extraction of EPS and Determination of EPS Concentrations

In this study, EPS indicated the substances extracted from activated sludge. To obtain EPS from the natural water, activated sludge was sampled from the Tsinghua pool in the near-term experiment. The 20 mL sludge mixture was removed from the reactor and centrifuged at 3000× *g* rpm for 10 min. Subsequently, EPS were extracted through the formaldehyde-NaOH extraction method [17]. The same 20 mL sludge mixture was transferred into a 50 mL centrifuge tube and added into 0.12 mL formaldehyde solution at 4 °C for 1 h. Then, 8 mL NaOH (1 mol·L^−1^) was added to the mixture and placed at 4 °C for 3 h. The mixture was centrifuged at 12,000× *g* for 20 min and the EPS was obtained after the supernatant was filtered with 0.22 μm filter membrane.

In order to accurately obtain the concentration of polysaccharides in EPS, a colorimetric method proposed by Dubois was adopted [18]. Every polysaccharide sample was hydrolyzed into furfural or hydroxymethyl glycolaldehyde under the action of concentrated sulfuric acid, and then condensed stably with phenol into a carmine compound [19]. Within a certain concentration range, the absorbance has a linear relationship with the concentration of total polysaccharides (glucose equivalents). By measuring the absorbance in 490 nm, the concentration of polysaccharides in the solution was converted by a standard curve. Meanwhile, by using Lowry’s method, UV-Vis spectrophotometer (Shimadzu UV-2501PC, Kyoto, Japan) took bovine serum albumin as the internal standard substance and made a quantitative test of protein [20]. Finally, the total amount of polysaccharides and proteins measured represented the content of EPS.

### 2.3. Filtration Tests

The commercial polyethersulfone ultrafiltration membranes were soaked in ultrapure water for at least 12 h to remove impurities. Membranes were stored at 4 °C before the filtration tests. In the following, the crossflow module of constant pressure at 2 bars was conducted in a 2 L plastic container and circulated at the speed of 10 cm/s. Lastly, the filtration time was controlled at 120 min and the change of effluent every 15 s were recorded in the computer connected with the electronic balance. The solvent permeation rate through the membrane can be calculated by Darcy’s Law (as shown in Equation (1)) [8]. According to the filtration test, the total filtration resistance (*R_t_*, m^−1^) can be determined. By using the resistance-in-series model, cleaning membrane resistance (*R_m_*), pore-blocking resistance (*R_p_*), and gel layer resistance (*R_g_*) were calculated by Equations (2) and (3):

(1) $$J=\frac{\Delta p}{\mu R}$$

(2) $$R_{t}=R_{m}+R_{p}+R_{c}$$

(3) $$R_{f}=R_{m}+R_{p}$$

where *J* is the effluent flux (LMH, m^3^·m^−2^·s^−1^); ∆p is the transmembrane pressure (TMP, Pa); μ is the solution viscosity (Pa·s).

### 2.4. Measurements of Adsorption Forces between Polysaccharides

Quartz-crystal microbalance with dissipation monitoring (QCM-D, Biolin Scientific, Gothenburg, Sweden) is an instrument that can real-time monitor the minute changes in mass adsorbed on a surface, which is used for analyzing the effect of antiscalants on bacterial deposition and attachment [21]. The adsorption degree of polysaccharides in solution was evaluated by QCM-D. The resonant frequency of the crystal will decrease with the deposition of the foulant on the membrane. QCM-D measurements were performed with a QSense Initiator system (Biolin Scientific, Sweden) and gold-coated quartz crystal sensors (Biolin Scientific, Sweden) with a fundamental resonance frequency of 4.95 MHz (C_QCM_, 17.77 ng·cm^−2^·Hz^−1^). Considering the importance of divalent cations in the spatial conformation of polysaccharides, Ca^2+^ and Mg^2+^ were selected as the basal solution and the change of adsorption quality was recorded to reflect the adsorption capacity of foulant [10]. All working solutions were performed by using a digital peristaltic pump with a 150 µL/min flow rate. The order of injection was as follows: (I) ultrapure water baseline for 20 min; (II) cationic solution for 20 min (control group); (III) alginate for 10 min; (IV) xanthan gum for 10 min; (V) SDS was used as a surfactant for 10 min to clean the organic foulant that had deposited; (VI) other steps were repeated in the same way. The data generated by Q-Tools software was exported and redrew. The ΔF and ΔD were measured for the 3rd overtone, and each sample was repeated at least three times. When organic foulant deposits on the gold-plated quartz sensor, the resonant frequency of the crystal will decrease and the amount of organic foulant macromolecule adsorbed can be calculated from the frequency change using the Sauerbrey equation (Equation (4)) [22,23].

(4) $$\Delta m=-\frac{C_{QCM}}{n}\Delta F$$

where *C_QCM_* is the mass sensitivity constant and is independent of the overtone number (*n*).

### 2.5. The Autopsy of Fouled Membranes

The fouling layer formed on the membrane surface was observed by scanning electron microscopy (SEM, ZEISS Sigma 500, Carl Zeiss, Oberkochen, Germany). Previously, the membrane foulants formed by crossflow filtration were pre-frozen at 4 °C for at least 24 h in the refrigerator and freeze-dried by lyophilizer (SCIENTZ-10N, Ningbo Xinzhi Biotechnology Co., Ltd., Ningbo, China). Subsequently, the membrane was sprayed with Pt for 30 s in an ion sputtering apparatus, and the surface morphology of fouled membrane was observed by SEM.

### 2.6. The Application of SVM in Fouling Analysis

SVM based on structural risk minimization is a machine learning method suitable for small-scale data sets. By means of a nonlinear mapping ϕ(x), SVM can make the linearly indivisible sample set linearly separable in a higher-dimensional space [24]. The traditional SVM algorithm is mainly aimed at binary classification problems (Equation (5)) [25]. In this study, the one-against-one strategy was used to solve the multi-category classification problem. The strategy was to construct a binary SVM between each of the two classes. In brief, for the *i*th and the *j*th classes, binary SVM solves the following quadratic programming problem:

$$\underset{w^{ij},b^{ij},\xi^{ij}}{\min}\;\frac{1}{2}{\left(w^{ij}\right)}^{T}w^{ij}+C\underset{t}{\sum }\xi^{ij}$$

$$\mathrm{subject}\;\mathrm{to}\;{\left(w^{ij}\right)}^{T}\phi \left(x_{t}\right)+b^{ij}\geq 1-\xi_{t}{}^{ij},\;if\;y_{t}=i$$

$${\left(w^{ij}\right)}^{T}\phi \left(x_{t}\right)+b^{ij}\leq -1+\xi_{t}{}^{ij},\;if\;y_{t}=j$$

(5) $$\xi_{t}{}^{ij}\geq 0$$

where C>0 is the regularization parameter, $x_{t}$ is the input sample vector and $y_{t}$ is the decision function for the *t*th sample of this binary SVM. For the convenience of calculation, the quadratic programming problem with high dimensional vector $w^{ij}$ is often transformed into its dual problem for solving. Accordingly, the following decision function can be obtained:

(6) $$y^{ij}\left(x\right)=\mathrm{sgn}\left[{\left(w^{ij}\right)}^{T}\phi \left(x\right)+b^{ij}\right]=\mathrm{sgn}\left[\overset{t^{ij}}{\underset{t=0}{\sum }}y_{t}\alpha_{t}^{ij}K\left(x_{t},\;x\right)+b^{ij}\right]$$

where $t^{ij}$ represents the sample size of this binary SVM, $0\leq \alpha_{t}^{ij}\leq C$, and $K\left(x_{t},\;x\right)$ is the kernel function. In this study, the commonly used Gaussian radial basis kernel function (RBF) was chosen as the kernel function of SVM [26]:

(7) $$K\left(x_{t},\;x\right)=\exp\left(-\frac{\|x_{t}-{\mathrm{x\|}}^{2}}{\sigma^{2}}\right)$$

Each trained SVM uses a voting strategy to make decisions on the classification result [27]. Votes for each class are counted, and the one with the largest number of votes is the predicted class. If the votes are tied, the class with a large sample size will simply be selected as the predicted. Following is the final decision function:

(8) $$f\left(x\right)=\underset{i=1,\cdots ,N}{\arg\;\max}\left[\overset{N}{\underset{j\neq i,j=1}{\sum }}y_{ij}\left(x\right)\right]$$

## 3. Results and Discussion

### 3.1. Gel Layer Formation: A Crucial Fouling Mechanism during Membrane Filtration Process

**Figure 1:**
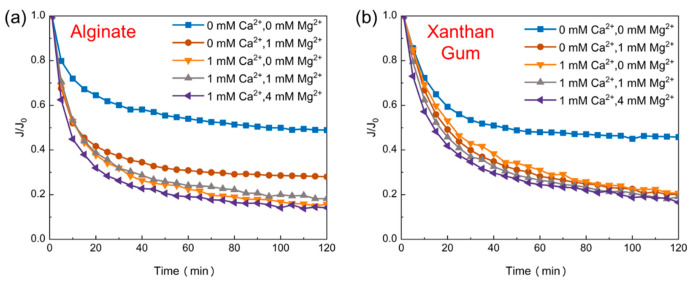
Filtration behaviors of (**a**) alginate and (**b**) xanthan gum (50 mg/L) in the presence of divalent cations.

The fouling propensities of typical polysaccharides (alginate and xanthan gum) were examined at the mere or concurrent presence of calcium ions and magnesium ions [28]. As shown in Figure 1a, calcium ions (1 mM) cause more serious membrane fouling than the same concentration of magnesium ions. Meanwhile, sodium alginate is significantly affected by calcium ions rather than magnesium ions in the coexistence of cations. Differently, as shown in Figure 1b, the permeable flux of xanthan gum is interfered by cations, but the concentration and variety of cations barely affects the fouling potential. Previous studies have shown that the presence of cations is the key factor that causes the conformational transition of xanthan gum in the solution which leads to the decline of permeation flux with the addition of cations [29]. On the other hand, xanthan gum has ion-sensitive characteristics and reaches saturation at very low concentrations of the divalent cations. Thus, the changes of the cation concentration and composition do not result in different fouling. Alginate is significantly affected by cation types, while xanthan gum is not, implying different filtration behaviors between alginate and xanthan gum with the presence of cations (Figure 1). Therefore, these two exopolysaccharides, when in natural water, have different fouling characteristics. In previous studies, our group defined them as “alginate-like” and “xanthan gum-like” for the mechanisms of the gel layer and intermediate blocking [8]. Furthermore, the coupled fouling properties of these two different polysaccharides should be discussed.

**Figure 2:**
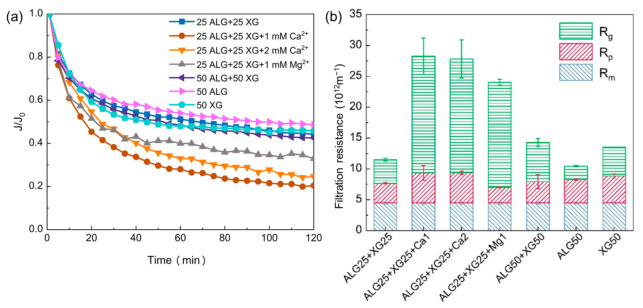
(**a**) The filtration behaviors and (**b**) distribution of resistance of mixed polysaccharides (mg/L) with the presence of divalent cations.

Figure 2a shows the effects of divalent cations on mixed polysaccharide aggregation. With the change of polysaccharide concentration from 50 mg/L to 100 mg/L, the filtration resistance appeared as a slight increase. Compared with the change of polysaccharide concentration, the presence of cations caused more flux decline and more serious membrane fouling. In order to figure out the main cause of fouling, and further predict the fouling potential of a certain feed, the resistance-in-series model was employed for the analysis of filtration behaviors of mixed polysaccharides (Figure 2b). At 50 mg/L total concentration of polysaccharide without cations, the fouling potential of mixed polysaccharide (ALG25 + XG25) was between mere alginate (ALG50) and xanthan gum (XG50), indicating that the possible interaction between alginate and xanthan does not greatly affect the fouling development. However, the resistance caused by the gel layer (R_g_) dramatically increased with the presence of cations. As discussed above, the fouling propensity of xanthan gum is not sensitive to the presence of cation. Furthermore, the adsorption capacity of alginate and xanthan gum on the membrane surface was evaluated by QCM-D and the results are shown in Table 2. With the presence of cations, the adsorption capacity of alginate and xanthan gum increased compared to that without cations. Whether in the presence of calcium ion or magnesium ion, alginate has a higher adsorption mass than xanthan gum. These results are inconsistent with the above filtration behaviors of the polysaccharides mixture, mainly, that alginate is more sensitive to cations. More importantly, as can be seen in Figure 2b, with the presence of cations in the polysaccharides mixture, gel layer formation is the main fouling mechanism. Therefore, the effect of cations on mixed polysaccharides is more likely due to the presence of alginate, which may play a leading role in the fouling potential of mixed polysaccharides.

### 3.2. Membrane Filtration Tests with the EPS and Surrogate Polysaccharides

**Figure 3:**
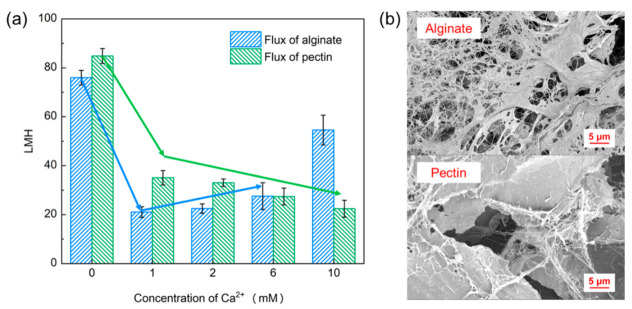
(**a**) The flux at the equilibrium of pectin and alginate (50 mg/L) with a series concentration of Ca^2+^; (**b**) the morphology characteristics of membrane surface formed by alginate and pectin after 120 min filtration test.

Some studies have shown that the gel layer formed on the membrane surface takes the main responsibility in polysaccharide fouling [8,30]. The specific properties, especially the permeabilities of different gel layers, should be explored. In this study, pectin and alginate, both of which mainly contribute to fouling by the formation of gel layer during membrane filtration, were employed to investigate the detailed fouling mechanisms [8]. With the different concentrations of calcium ion, the results of filtration tests of alginate and pectin are shown in Figure 3a. Consistent with previous studies, the fouling propensities of alginate first increased and then decreased alongside the increasing concentration of calcium ion [10,28]. Calcium ion at 1 mM would cause the most serious fouling phenomenon. The membrane fouling of pectin continuously aggravated with the addition of more calcium ion. The calcium ion at high concentration may work in a similar fashion as coagulant in alleviating alginate fouling [10]. However, the pectin has a totally different binding mechanism with calcium ion due to its methylation [31]. At a low ion concentration of Ca^2+^ (1 mM), the interaction between pectin and calcium ion is established by hydrogen bonds [32], which leads to the extremely unstable and loose pectin gel. With an increasing of the calcium ions level, the strength and hardness of the gel enhance as the water-holding capacity of pectin decreases [33]. As a consequence of this, the gel layer formed at high concentration of Ca^2+^ shows a high filtration resistance as can be observed in Figure 3a. Furthermore, as is shown in Figure 3b, the membrane surfaces fouled by alginate and pectin are observed by SEM. It shows the different morphologic characteristics of gel layers formed by alginate and pectin with the presence of 1 mM calcium ion [8]. Recently, researchers reported the substantial differences in the colloid properties and membrane fouling behaviors between alginate and EPS, and thusly, alginate is not a perfect surrogate for practical feed water to explore fouling mechanism [11,15]. Therefore, pectin is analyzed to reflect the fouling potential of EPS instead of alginate.

**Figure 4:**
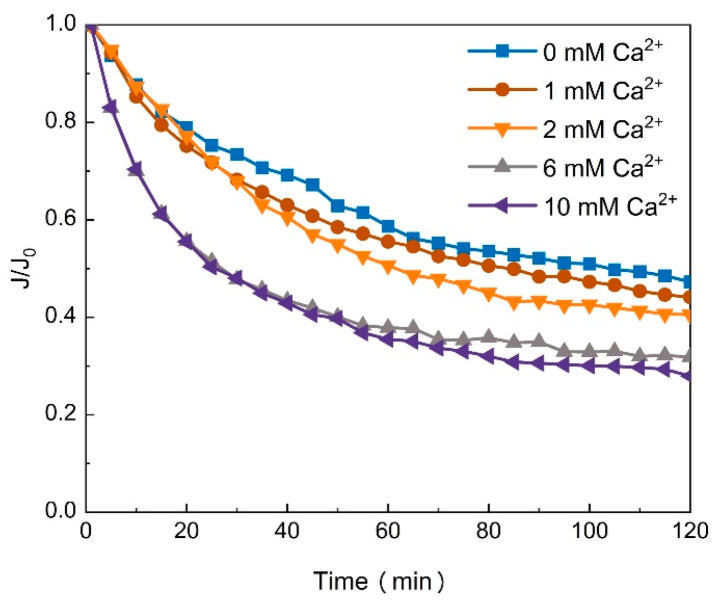
The filtration behaviors of EPS with the addition of a series of concentrations of Ca^2+^.

Previous studies have shown that the main composition of gel layer adhered to the surface of membrane was EPS produced from germ in activated sludge [34]. After the determination, the concentrations of EPS in solution are 270 mg/g VSS (volatile suspended solids), which may promote the formation of gel layers. As shown in Figure 4, with the addition of more calcium ions, the fouling potential of EPS increased, consistent with the results of pectin (Figure 3a). As mentioned above, alginate has the same fouling mechanism as pectin except for the influence of cations. Meanwhile, with the change of cations, EPS has the same fouling mechanism [35] and filtration behaviors as pectin. Therefore, as a novel surrogate, pectin may be applied to the fouling studies of EPS when alginate is not applicable.

### 3.3. The Implications for Analysis of EPS Fouling

As major foulants among natural organic matter, polysaccharides can cross-link or combine with other organic molecules to form a three-dimensional network structure [6,7]. Thus, polysaccharides frequently contribute to irreversible membrane fouling and play a more important role than other organic foulants in membrane fouling. The dominant factors of polysaccharide fouling are determined by its specific functional groups, spatial conformations of chains, and environmental conditions (temperature, pH, ionic strength, etc.) [36]. In addition, EPS contains more organic matter, and alginate employed to represent all polysaccharides in fouling studies, is not perfectly representative. For example, in this work, it shows that the similarity between pectin and extracted EPS is higher. Moreover, the composition and content of EPS depend on the source of feed water. Therefore, the fouling potential of various EPS should be explored. In addition, the components of EPS are greatly influenced by the different extraction methods. At present, the extraction methods of EPS include heating, ultrasonic, formaldehyde-NaOH, and ethylene diamine tetraacetic acid (EDTA) addition. Among them, the formaldehyde-NaOH extraction method has the highest extraction efficiency for EPS [17]. Different extraction methods should be considered in investigation of fouling problems.

### 3.4. Application of SVM to Predict the Type of Membrane Fouling

As a newly developed technique, SVM is usually capable of reaching better accuracy of classification with limited samples. Therefore, the fouling modeling, based on SVM for identifying the fouling mechanisms of unknown feed water and for seeking more appropriate surrogate polysaccharides, has been established [15]. As shown in Table 3, according to the previous classification, five polysaccharides were selected as data sets for training. One hundred and two data sets (filtration curve similar to Figure 1) were extracted from published studies, and this paper divided them into two categories: learning and validation [8]. To minimize the impact brought by imbalanced sample numbers among types, different weights were placed on each type, according to the sample number in the algorithm. Generally, 80% of the collected data was used for training and establishing the discriminant ability of the SVM. Meanwhile, the remaining 20% was used for validation.

The SVM algorithms were coded and developed in MATLAB R2019a (The Math Works Inc., Natick, Massachusetts, USA). The CPU of the PC is Intel (R) Core (TM) i7-7700, and the RAM is 16 GB. Meanwhile, to evaluate the performance of SVM in the type of membrane blocking, one statistical parameter was considered: accuracy(A). The confusion matrix (Table 4) makes it easy to evaluate the performance of multi-class data sets. According to the confusion matrix, the accuracy is calculated as follow:

(9) $$\mathrm{Accracy}=\frac{\sum_{i=1}^{5}P_{ii}}{N}$$

**Figure 5:**
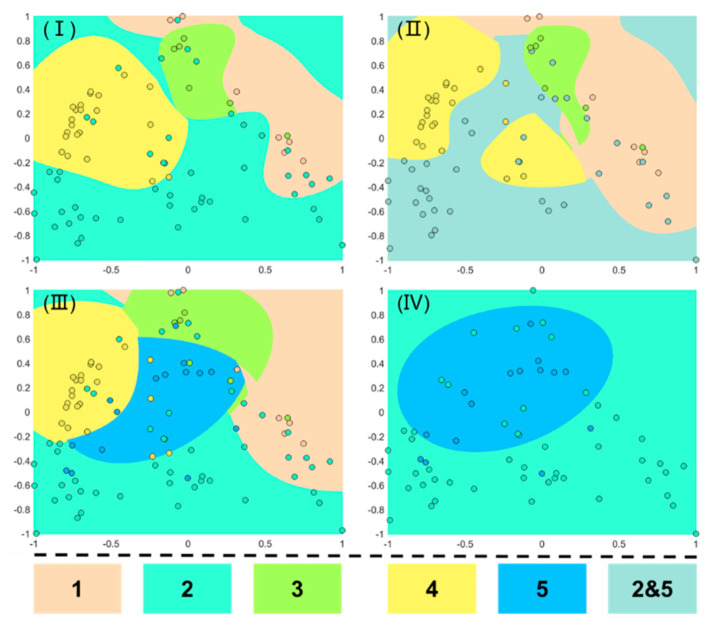
The simulation results of categories and accuracy of training with SVM (the accuracy of training: (**I**) tranching: 1, 2, 3, 4 (68.42%); (**II**) tranching: 1, 2 and 5, 3, 4 (72.73%); (**III**) tranching: 1, 2, 3, 4, 5 (59.09%); (**IV**) tranching: 2, 5 (76.92%)). (The meaning of the numbers is shown in Table 3).

Lastly, as shown in Figure 5, four classified calculation methods were performed to validate the accuracy of SVM as well as the possibility of pectin as a surrogate for EPS. Each of the four categories involves k(k − 1)/2 binary SVM. Compared with group I, group II and III supplement the training set of pectin. As the fouling mechanism of the gel layer, alginate and pectin were confirmed to have certain differences with the presence of cations. As mentioned above, pectin may be more appropriate as a novel surrogate than alginate to be applied in the studies of EPS fouling. “Identification” represents the degree of distinction between the four fouling mechanisms, or the validation accuracy of identifying the fouling mechanism of unknown feed water. As shown in Figure 5, the validation accuracy of SVM on membrane fouling is 68.42% when only four polysaccharides are employed to represent fouling mechanisms. As pectin was added to the database of gel layer, the accuracy was improved to 72.73%. This indicated that SVM had been successfully applied in recognizing the fouling mechanism, and the validation accuracy will be improved with an increase in foulant surrogate. However, pectin listed as a separate category will greatly increase the difficulty of SVM validation. Therefore, two-step recognition was introduced to identify the different mechanisms of the gel layer. As is shown in group IV, the SVM algorithms can meet the requirements of partition to a great extent. The results indicated that SVM can identify the differences (76.92%) between alginate and pectin with the presence of cations in the fouling mechanism of gel layer formation. Meanwhile, the identification result of filtration behaviors of EPS was consistent with pectin, which indicated the reliability of pectin working as the EPS surrogates (76.92%). As such, the database of SVM training based on mathematical models and practical parameters can be obtained to identify the fouling mechanisms of unknown feed water to the membrane system [15]. Thus, SVM may be a more powerful tool to predict complex filtration behaviors than traditional empirical models.

## 4. Conclusions

In this study, the fouling mechanisms of classic foulant surrogates (alginate and xanthan gum) were systematically studied and results showed that the alginate fouling is more sensitive to cations than xanthan. Furthermore, filtration tests with mixtures of alginate and xanthan revealed that alginate may play a leading role in the fouling potential. In addition, EPS was extracted from the sludge by the formaldehyde-NaOH extraction method, and the filtration behaviors of alginate, pectin, and EPS were examined. As a novel surrogate foulant, pectin seems to share more similarities in fouling with EPS, while alginate does not. In order to further analyze the fouling mechanisms, this study proposes an SVM modeling process based on the performances of filtration. By establishing the optimal parameters and using the strategy model classification, the recognition system of foulant surrogates based on SVM was designed and accomplished. It proved that, technically, the SVM can provide an excellent simulation to recognize the foulant surrogates of the practical feed water. In future work, more fouling data should be involved in the database in order to improve the performance of this SVM model, and thus to respond to the complexity of various feed water to membrane systems.

## Figures and Tables

**Table 1:** The concentration parameters of the sample solution.

	Polysaccharides (mg/L)	Ca^2+^ (mM)	Mg^2+^ (mM)
A	Alginate (50 mg/L)	0	0, 1
		1	0, 1, 4
	Xanthan gum (50 mg/L)	0	0, 1
		1	0, 1, 4
B	Alginate and xanthan gum (25 mg/L, respectively)	0	0, 1
		1	0
		2	0
	Alginate and xanthan gum	0	0
C	Alginate (50 mg/L)	0, 1, 2, 6, 10	0
	Pectin (10, 50 mg/L)	0, 1, 2, 6, 10	0

**Table 2:** Adsorption mass of alginate and xanthan gum deposited on the gold-coated quartz crystal sensors (ng/cm^2^).

Polysaccharide	Without Cations (ng)	Mg^2+^ (ng)	Ca^2+^ (ng)
Alginate	0.4754 ± 0.0292	5.8891 ± 0.0652	5.9683 ± 0.0597
Xanthan gum	0.2066 ± 0.0330	2.0566 ± 0.0764	2.2125 ± 1.4847

**Table 3:** Five representative polysaccharides to reflect different mechanisms of membrane fouling [8].

Number	Model	Polysaccharide
1	Standard blocking	Agarose (AG)
2	Gel	Alginate (ALG)
3	Complete blocking	Starch (S)
4	Intermediate blocking	Xanthan gum (XG)
5	Gel-like	Pectin (P)

**Table 4:** Confusion matrix (taking the five classes as examples).

Correct Class	Predicted Class					
	AG (1)	ALG (2)	S (3)	XG (4)	P (5)	Total
AG (1)	$P_{11}$	$P_{12}$	$P_{13}$	$P_{14}$	$P_{15}$	$T_{l1}$
ALG (2)	$P_{21}$	$P_{22}$	$P_{23}$	$P_{24}$	$P_{25}$	$T_{l2}$
S (3)	$P_{31}$	$P_{32}$	$P_{33}$	$P_{34}$	$P_{35}$	$T_{l3}$
XG (4)	$P_{41}$	$P_{42}$	$P_{43}$	$P_{44}$	$P_{45}$	$T_{l4}$
P (5)	$P_{51}$	$P_{52}$	$P_{53}$	$P_{54}$	$P_{55}$	$T_{l5}$
Total	$T_{c1}$	$T_{c2}$	$T_{c3}$	$T_{c4}$	$T_{c5}$	N

## Data Availability

All the data supporting the findings of this study are available within the article.
